# Supplementary material for: Prognostic Value of Blood-Based Inflammatory Markers in Cancer Patients Receiving Immune Checkpoint Inhibitors
Source: Cancers (Basel). 2024 Dec 26;17(1):37. doi: 10.3390/cancers17010037 (PMC11719015; doi:10.3390/cancers17010037)
Supplement: Supplementary file 1 [file cancers-17-00037-s001.zip › cancers-3350725-supplementary.pdf]

Supplementary Table S1. Summary of baseline blood counts & blood-based inflammatory markers of the study population

	x̄±SD	Median (Min-Max)
**Leucocyte (10^3/µL)**	7543.89 ± 2834.15	7245 (2530-23230)
**Neutrophile (10^3/µL)**	4868.98 ± 2379.79	4415 (800-18050)
**Lymphocyte (10^3/µL)**	1785.49 ± 853.58	1635 (330-6110)
**Eosinophile (10^3/µL)**	168.11 ± 130.37	130 (0-670)
**Monocyte (10^3/µL)**	691.68 ± 314.06	545 (60-2380)
**Platelet (10^3/µL)**	281035.4 ± 112588.8	263500 (36000-676000)
**Hemoglobin (g/dL)**	12.2 ± 2.05	12.3 (6.6-17.1)
**CRP (mg/L)**	27 ± 38.4	11 (0.3-220)
**Albumin (g/L)**	40.8 ± 4.9	41 (23-51)
**LDH (U/L)**	263.8 ± 178.2	210.5 (109-1746)
**NLR**	3.3 ± 2.2	2.9 (0.36-12.9)
**ELR**	0.1 ± 0.1	0.09 (0-0.65)
**PIV**	809 ± 1252	451.8 (5.68-12800)
**SII**	992.3 ± 978.6	676.9 (20.3-7664.8)
**SIRI**	2.5 ± 2.7	1.8 (0.097-21.6)

ELR, Eosinophil/Lymphocyte Ratio; Hb, Hemoglobin; LDH, Lactate Dehydrogenase; NLR, Neutrophil /Lymphocyte Ratio;PIV, Pan-Immune-Inflamation Value; SII, Systematic Immune-Inflamation Index; SIRI, Systematic Inflamatory Response Index

Figure S1. Heatmap of the correlation coefficients of inflammatory markers (correlation coefficient R, form −1 [blue] to 1 [yellow], *P* < 0.01 was labeled as *)

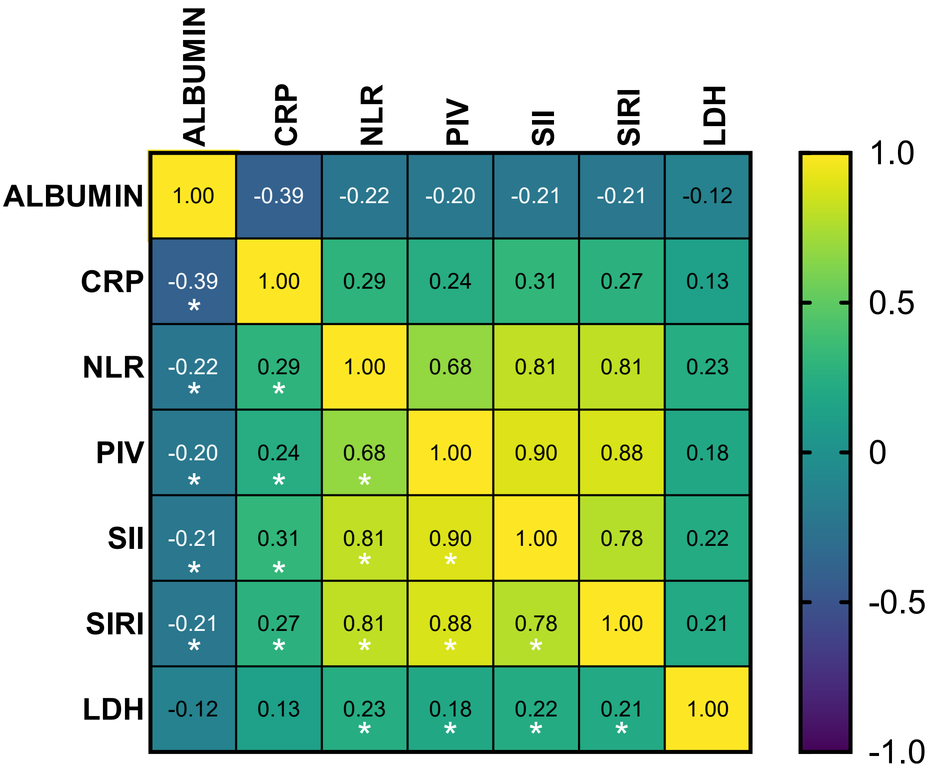

Supplementary Table S2. Correlation Matrix

		ALBUMIN	CRP	NLR	PIV	SII	SIRI
CRP	Pearson's r	-0.390	—				
	p-value	< .001	—				
NLR	Pearson's r	-0.219	0.287	—			
	p-value	< .001	< .001	—			
PIV	Pearson's r	-0.205	0.240	0.678	—		
	p-value	0.002	0.002	< .001	—		
SII	Pearson's r	-0.213	0.314	0.813	0.898	—	
	p-value	0.001	< .001	< .001	< .001	—	
SIRI	Pearson's r	-0.212	0.275	0.809	0.883	0.779	—
	p-value	0.001	< .001	< .001	< .001	< .001	—
LDH	Pearson's r	-0.115	0.127	0.234	0.184	0.222	0.207
	p-value	0.090	0.114	< .001	0.006	< .001	0.002
